# Supplementary material for: Anchor objects drive realism while diagnostic objects drive categorization in GAN generated scenes
Source: Commun Psychol. 2024 Jul 26;2:68. doi: 10.1038/s44271-024-00119-z (PMC11332195; doi:10.1038/s44271-024-00119-z)
Supplement: Supplementary file 2 — Supplementary Information [file 44271_2024_119_MOESM2_ESM.pdf]

Supplementary Information

Supplementary Figure 1. Sensitivity (D’) and Bias Experiment 1. Participants performed a two alternative forced-choice task (2AFC) where they were instructed to discriminate between real and generated images in two presentation time conditions (50 ms and 500 ms). In the main analysis we modelled responses from a set of predictors in a generalized linear mixed model (glmm) where interactions with the true image condition reflect effects on sensitivity (D’). Here, we plot overall sensitivity and bias across both presentation time conditions. D’ 50 ms: M = 0.53, SE = 0.06; D’ 500 ms: M = 1.95, SE = 0.17. Gray points and lines represent individual participants (N=50), black line and points represent average across participants.

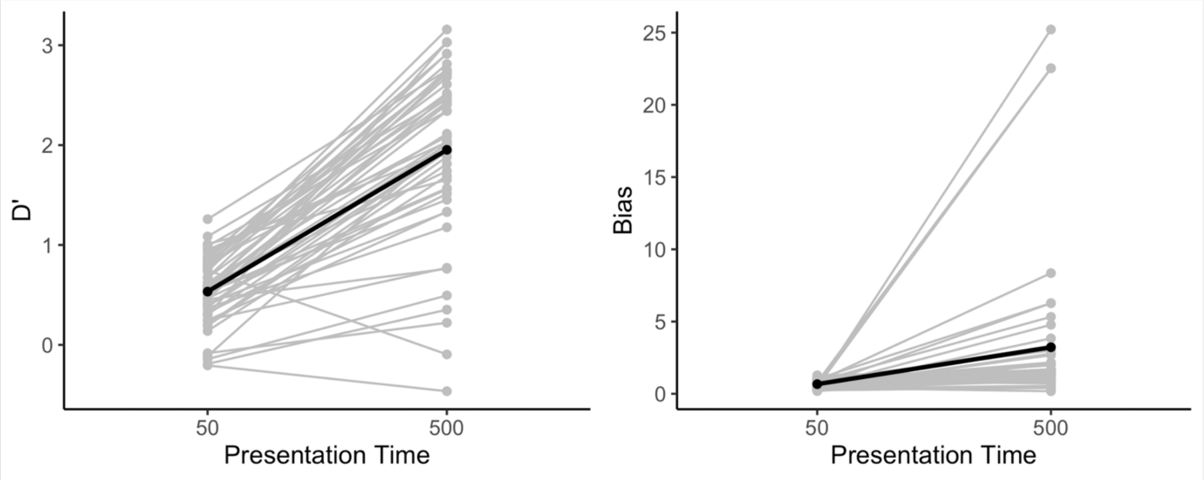

Supplementary Figure 2. High-level visual features examples. We use two common deep neural network (DNN) visualization techniques: Grad-Cam[1] and class specific image generatation [2,3] to provide a better intuition for high-level visual features in DNNs. Grad-Cam produces 'visual explanations' for DNN predictions. Given some image and a pretrained convolutional DNN Grad-Cam returns a coarse localitation map for important regions in the image regarding one of the trained concepts. Class specific image generation is a set of methods implementing regularized optimization in image space, that is generating an image which maximises the score for one particular class. In the paper we use a range of DNNs, for the purpose of these exemplary visualisations we apply both techniques to a basic pre-trained convolutional DNN AlexNet[4] trained on the Places365 image database (365-way scene classification). We use code from [5] and generated images used in the present study to produce the visualisation below.

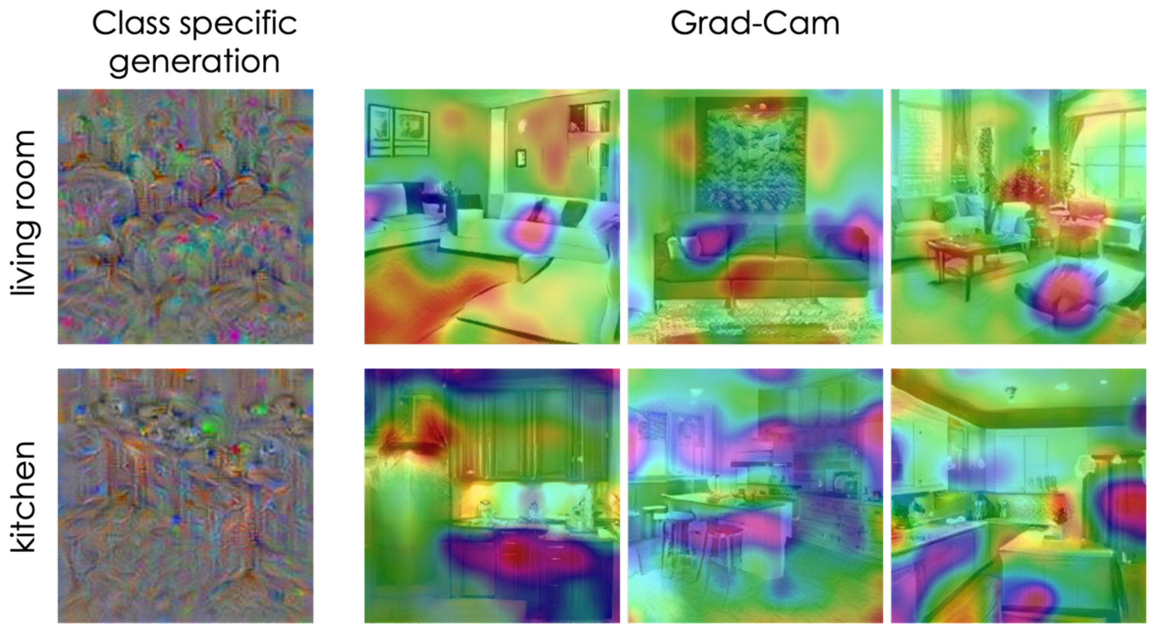

Supplementary Table 1

Outcome of model comparison (Experiment 1, 2AFC task).

Model	GLMM Model Formula	AIC	Model for Bayes Factor analysis	BF	% error
Full model	Realness response (1=real/0=generated) ~ true image condition*presentation duration*anchor status frequency*diagnosticity+ (1+true image condition+ presentation duration+ diagnosticity | participant)+ (1+presentation duration | stimulus)	13402	M_f: Realness response (1=real/0=generated) ~ true image condition+presentation duration+anchor status frequency+diagnosticity+ true image condition:presentation duration+true image condition:diagnosticity	BF_{f0}: 4.37×10^{1339}	1.76
Null model	Realness response (1=real/0=generated) ~ 1+ (1+true image condition+ presentation duration+ diagnosticity | participant)+ (1+presentation duration | stimulus)	13696	M_0: Realness response (1=real/0=generated) ~ 1		
Model without diagnosticity	Realness response (1=real/0=generated) ~ true image condition*presentation duration*anchor status frequency + (1+true image condition+ presentation duration | participant) + (1+presentation duration | stimulus)	13395	*M_1: Realness response (1=real/0=generated) ~ true image condition+presentation duration+anchor status frequency+true image condition:presentation duration+true image condition:diagnosticity	BF_{10}: 1.75×10^{1340}<br>BF_{1f}: 4	1.65<br>2.41
Model without anchor status frequency	Realness response (1=real/0=generated) ~ true image condition+presentation duration+diagnosticity+true image+condition:presentation duration+true image condition:diagnosticity	13398	M_2: Realness response (1=real/0=generated) ~ true image condition+presentation duration+true image condition:presentation duration+true image condition:diagnosticity	BF_{20}: 1.26×10^{1335}<br>BF_{f2}: 3.46×10^{4}	1.34<br>2.21

Note. Bayes factors were computed for each model and a null model using default mixture-of-variance priors[6–8] and for individual effects using subscripts to indicate the direction of the comparison. The null model was a model with an additive model on the random factor (participant) plus intercept (grand mean). All models include participant. Most preferential model based on BF analysis comparing all possible sub-models of M_f is marked with (*)

Supplementary Table 2

Outcome of model comparison (Experiment 1, rating task).

Model	LMM Model Formula	AIC	Model for Bayes Factor analysis	BF	% error
Full model	Realness rating (1-6) ~ anchor status frequency*diagnosticity+ (1 | participant)+ (1 | stimulus)	25061	*M_f: Realness rating (1-6) ~ anchor status frequency*diagnosticity	BF_{f0}: 1.65×10^{564}	0.21
Null model	Realness rating (1-6) ~ 1+ (1 | participant)+ (1 | stimulus)	25061	M_0: Realness rating (1-6) ~ 1		
Model without diagnosticity	Realness rating (1-6) ~ anchor status frequency+ (1 | participant)+ (1 | stimulus)	25060	M_1: Realness rating (1-6) ~ anchor status frequency	BF_{10}: 6.45×10^{556}<br>BF_{f1}: 2.56×10^{7}<br>BF_{12}: 1×10^{5}	0.78<br>0.81<br>0.92
Model without anchor status frequency	Realness rating (1-6) ~ diagnosticity+(1 | participant)+ (1 | stimulus)	25062	M_2: Realness rating (1-6) ~ diagnosticity	BF_{20}: 6.64×10^{551}<br>BF_{f2}: 2.49×10^{12}	0.49<br>0.54

Note. Bayes factors were computed for each model and a null model using default mixture-of-variance priors[6–8] and for individual effects using subscripts to indicate the direction of the comparison. The null model was a model with an additive model on the random factor (participant) plus intercept (grand mean). All models include participant. Most preferential model based on BF analysis comparing all possible sub-models of M_f is marked with (*)

Supplementary Table 3

Outcome of model comparison (Experiment 2, 5AFC task).

Model	GLMM Model Formula	AIC	Model for Bayes Factor analysis	BF	% error
Full model	Categorization accuracy (1=accurate/0=inaccurate) ~ true image condition+presentation duration+realness+diagnosticity+anchor status frequency)^4+ (1+true image condition+ presentation duration | participant)+ (1+presentation duration | stimulus)	7983	*M_f: Categorization accuracy (1=accurate/0=inaccurate) ~ true image condition+ realness+presentation duration+diagnosticity+ anchor status frequency	BF_{f0}: 1.26x10^{263}	0.74
Null model	Categorization accuracy (1=accurate/0=inaccurate) ~ 1+ (1+true image condition+presentation duration | participant)+(1+presentation duration | stimulus)	8072	M_0: Categorization accuracy (1=accurate/0=inaccurate) ~ 1		
Model without diagnosticity	Categorization accuracy (1=accurate/0=inaccurate) ~ true image condition+ presentation duration+ realness+anchor status frequency)^4+ (1+true image condition+ presentation duration | participant)+ (1+presentation duration | stimulus)	7983	M_1: Categorization accuracy (1=accurate/0=inaccurate) anchor status frequency+ true image condition+ realness+presentation duration+anchor status frequency	BF_{10}: 2.06x10^{239}<br>BF_{f1}: 6.08x10^{23}<br>BF_{21}: 4.33x10^{22}	1.19<br>1.4<br>1.42
Model without anchor status frequency	Categorization accuracy (1=accurate/0=inaccurate) ~ true image condition+ presentation duration+realness+ diagnosticity)^4+ (1+true image condition+ presentation duration | participant)+ (1+presentation duration | stimulus)	7971	M_2: Categorization accuracy (1=accurate/0=inaccurate) ~ true image condition+ realness+presentation duration+diagnosticity	BF_{20}: 8.95x10^{261}<br>BF_{f2}: 14.03	0.78<br>1.07

Note. Bayes factors were computed for each model and a null model using default mixture-of-variance priors[6–8] and for individual effects using subscripts to indicate the direction of the comparison. The null model was a model with an additive model on the random factor (participant) plus intercept (grand mean). All models include participant. Most preferential model based on BF analysis comparing all possible sub-models of M_f is marked with (*)

Supplementary References

1. Selvaraju, R. R. *et al.* Grad-CAM: Visual Explanations from Deep Networks via Gradient-Based Localization. *Int J Comput Vis* **128**, 336–359 (2020) doi:10.1007/s11263-019-01228-7.
2. Simonyan, K., Vedaldi, A. & Zisserman, A. Deep Inside Convolutional Networks: Visualising Image Classification Models and Saliency Maps. Preprint at https://doi.org/10.48550/arXiv.1312.6034 (2014) doi:10.48550/arXiv.1312.6034.
3. Yosinski, J., Clune, J., Nguyen, A., Fuchs, T. & Lipson, H. Understanding Neural Networks Through Deep Visualization. Preprint at https://doi.org/10.48550/arXiv.1506.06579 (2015) doi:10.48550/arXiv.1506.06579.
4. Krizhevsky, A., Sutskever, I. & Hinton, G. E. ImageNet classification with deep convolutional neural networks. *Commun. ACM* **60**, 84–90 (2017) doi:10.1145/3065386.
5. Ozbulak, U. PyTorch CNN Visualizations. *GitHub repository* (2019).
6. Morey, R. D. & Rouder, J. N. *BayesFactor: Computation of Bayes Factors for Common Designs*. (2024).
7. Rouder, J. N. & Morey, R. D. Default Bayes Factors for Model Selection in Regression. *Multivariate Behavioral Research* **47**, 877–903 (2012) doi:10.1080/00273171.2012.734737.
8. Liang, F., Paulo, R., Molina, G., Clyde, M. A. & Berger, J. O. Mixtures of *g* Priors for Bayesian Variable Selection. *Journal of the American Statistical Association* **103**, 410–423 (2008) doi:10.1198/016214507000001337.
