# Supplementary material for: Anchor objects drive realism while diagnostic objects drive categorization in GAN generated scenes
Source: Commun Psychol. 2024 Jul 26;2:68. doi: 10.1038/s44271-024-00119-z (PMC11332195; doi:10.1038/s44271-024-00119-z)
Supplement: Supplementary file 3 — Reporting Summary [file 44271_2024_119_MOESM3_ESM.pdf]

nature portfolio

Corresponding author(s): Aylin Kallmayer

Last updated by author(s): Jul 8, 2024

Reporting Summary

Nature Portfolio wishes to improve the reproducibility of the work that we publish. This form provides structure for consistency and transparency in reporting. For further information on Nature Portfolio policies, see our Editorial Policies and the Editorial Policy Checklist.

Statistics

For all statistical analyses, confirm that the following items are present in the figure legend, table legend, main text, or Methods section.

n/a	Confirmed	
☐	☒	The exact sample size (n) for each experimental group/condition, given as a discrete number and unit of measurement
☐	☒	A statement on whether measurements were taken from distinct samples or whether the same sample was measured repeatedly
☐	☒	The statistical test(s) used AND whether they are one- or two-sided<br>*Only common tests should be described solely by name; describe more complex techniques in the Methods section.*
☐	☒	A description of all covariates tested
☐	☒	A description of any assumptions or corrections, such as tests of normality and adjustment for multiple comparisons
☐	☒	A full description of the statistical parameters including central tendency (e.g. means) or other basic estimates (e.g. regression coefficient) AND variation (e.g. standard deviation) or associated estimates of uncertainty (e.g. confidence intervals)
☐	☒	For null hypothesis testing, the test statistic (e.g. F, t, r) with confidence intervals, effect sizes, degrees of freedom and P value noted<br>*Give P values as exact values whenever suitable.*
☐	☒	For Bayesian analysis, information on the choice of priors and Markov chain Monte Carlo settings
☐	☒	For hierarchical and complex designs, identification of the appropriate level for tests and full reporting of outcomes
☐	☒	Estimates of effect sizes (e.g. Cohen's d, Pearson's r), indicating how they were calculated

Our web collection on statistics for biologists contains articles on many of the points above.

Software and code

Policy information about availability of computer code

Data collection	Psychopy3 v2023.1.0 and Pavlovia to host the experiments online. Psychopy builder files and stimuli are available via OSF
Data analysis	All data was pre-processed and analysed using the R statistical programming language (v4.1.2, R Core Team, 2021) in RStudio (v2.3.492).<br>Deep neural networks were deployed using Python v3.7.16

For manuscripts utilizing custom algorithms or software that are central to the research but not yet described in published literature, software must be made available to editors and reviewers. We strongly encourage code deposition in a community repository (e.g. GitHub). See the Nature Portfolio guidelines for submitting code & software for further information.

Data

Policy information about availability of data

All manuscripts must include a data availability statement. This statement should provide the following information, where applicable:

- Accession codes, unique identifiers, or web links for publicly available datasets
- A description of any restrictions on data availability
- For clinical datasets or third party data, please ensure that the statement adheres to our policy

Raw data and code, stimuli, experimental files (psychopy builder files) are available at the Open Science Framework: https://osf.io/x2rbq/?view_only=fbdb72f4a8904f9dae6d39d3e02f7cb5

Human research participants

Policy information about studies involving human research participants and Sex and Gender in Research.

Reporting on sex and gender	Sex and gender were not considered in the study design. Participants reported their gender online before the experiment started (experiment 1: 36 women, 14 men, 0 non-binary participants, 0 participants with undisclosed gender; experiment 2: 30 women, 14 men, 0 non-binary participants, 0 participants with undisclosed gender).
Population characteristics	Experiment 1 mean age = 20.74, SD = 2.5; Experiment 2 mean age = 23.2, SD = 5.3
Recruitment	Participants were recruited at random via SONA (https://uni-frankfurt.sona-systems.com/Default.aspx?ReturnUrl=%2f).
Ethics oversight	Informed consent was given via an online form. All aspects of data collection and analysis were carried out in accordance with guidelines approved by the Human Research Ethics Committee of the Goethe University Frankfurt.

Note that full information on the approval of the study protocol must also be provided in the manuscript.

Field-specific reporting

Please select the one below that is the best fit for your research. If you are not sure, read the appropriate sections before making your selection.

☐ Life sciences ☒ Behavioural & social sciences ☐ Ecological, evolutionary & environmental sciences

For a reference copy of the document with all sections, see nature.com/documents/nr-reporting-summary-flat.pdf

Behavioural & social sciences study design

All studies must disclose on these points even when the disclosure is negative.

Study description	We ran two online behavioral experiments
Research sample	Fifty participants completed Experiment 1 (36 women, 14 men, 0 non-binary participants, 0 participants with undisclosed gender, M = 20.74 years old, SD = 2.5) and 44 participants completed Experiment 2 (30 women, 14 men, 0 non-binary participants, 0 participants with undisclosed gender, M = 23.2 years old, SD = 5.3). Participants were recruited online via SONA (https://www.sona-systems.com) and received course credit for participation. Normal or correct-to-normal vision was stated as condition to participate, however, participants did not have to perform any tests prior to participation. Participants were unfamiliar with the stimulus material and could only participate once in either Experiment 1 or Experiment 2. Informed consent was provided via an online form before the experiments. All aspects of data collection and analysis were carried out in accordance with guidelines approved by the Human Research Ethics Committee of the Goethe University Frankfurt.
Sampling strategy	Prior simulation based power analyses suggested 50 participants for both experiments. Six participants had to be excluded from Experiment 2 because they aborted the online experiment before completing all trials.
Data collection	Participation occurred online. Smartphones or tablets were not permitted. Participants were instructed to seek a quiet environment where they could expect little distraction.
Timing	Experiment 1: November 2021; Experiment 2: March - June 2022, August-October 2022
Data exclusions	6 participants were excluded from experiment 2 as they did not complete the task properly (only completed a subset of trials before aborting the experiment)
Non-participation	The 6 participants that did not finish the experiment did not provide any reasons.
Randomization	Conditions were manipulated within participants and the allocation of condition to stimulus was counterbalanced between participants.

Reporting for specific materials, systems and methods

We require information from authors about some types of materials, experimental systems and methods used in many studies. Here, indicate whether each material, system or method listed is relevant to your study. If you are not sure if a list item applies to your research, read the appropriate section before selecting a response.

Materials & experimental systems

n/a	Involved in the study
☒	☐ Antibodies
☒	☐ Eukaryotic cell lines
☒	☐ Palaeontology and archaeology
☒	☐ Animals and other organisms
☒	☐ Clinical data
☒	☐ Dual use research of concern

Methods

n/a	Involved in the study
☒	☐ ChIP-seq
☒	☐ Flow cytometry
☒	☐ MRI-based neuroimaging
